# Supplementary material for: Dynamic transcriptomic profiles of zebrafish gills in response to zinc supplementation
Source: BMC Genomics. 2010 Oct 11;11:553. doi: 10.1186/1471-2164-11-553 (PMC3091702; doi:10.1186/1471-2164-11-553)
Supplement: Additional file 2 — Interactive Direct Interaction Network representing the molecular interactions between zinc, copper, iron, calcium and proteins encoded by transcripts changed by zinc supplementation. Mini web-site containing index.html and hyperlinked pages in subdirectory describing a Direct Interaction Network automatically generated based on curated interactions contained within the proprietary PathwayArchitect database. Ovals represent proteins and the circles symbolize metal ions. Objects are coloured by their abundance in zebrafish at the time-point they were significantly different from the control is a scale from -4 fold (dark green) to +4 fold (dark red). Where significant differences were found at more than one time-point, the colour overlay shows expression at the first instance. Dark blue squares denote 'binding', and light blue squares 'expression'; green squares stand for 'regulation', green diamonds for 'metabolism', and green circles for 'promoter binding'. Arrow heads indicate directionality of the interaction where annotated. All nodes and edges can be further interrogated by selecting the relative area of the image. [file 1471-2164-11-553-S2.zip › PathwayArchitect Zn xs DIN/481605.html]

# EXPRESSION:

|  |  |
| --- | --- |
| Type | EXPRESSION |
| Effect | None |


---

|  |  |
| --- | --- |
| Score | 0 |


---

|  |  |
| --- | --- |
| Reference Count | 11 |


---

|  |  |
| --- | --- |
| Mechanism | Unknown |


---

|  |  |
| --- | --- |
| Reference:0 || Sentence | "The effects of iron perturbations on DMT1 and FPN1 expression were investigated in CaCo2 cells and in primary tissue cultures of human duodenal biopsies by means of Northern Blot, Western Blot, RNA-bandshift and Nuclear Run off analysis.Both DMT1 and FPN1 mRNA levels were increased upon treatment of CaCo2 cells with desferrioxamine, whereas iron treatment resulted in the opposite effect." |
| PMID | 12547239 |
| Year | 2002 |
| Species | Human |
| Journal | Blood Cells Mol Dis |
| RefScore | 1 |
| Source | PArchNLP |
  |
|  |  |


---

|  |  |
| --- | --- |
 Reference:1 || Sentence | "Nuclear run-off analysis then demonstrated that the effects of iron and desferrioxamine on DMT1 and FPN1 mRNA expression are rather due to modulation of transcription of these genes." |
| PMID | 12547239 |
| Year | 2002 |
| Species | Human |
| Journal | Blood Cells Mol Dis |
| RefScore | 1 |
| Source | PArchNLP |
  ||  |  |


---

|  |  |
| --- | --- |
 Reference:2 || Sentence | "Our results demonstrate that iron unidirectionally regulates the expression of the two ferrous ion transporters DMT1 and FPN1 by affecting their transcription." |
| PMID | 12547239 |
| Year | 2002 |
| Species | Human |
| Journal | Blood Cells Mol Dis |
| RefScore | 1 |
| Source | PArchNLP |
  ||  |  |


---

|  |  |
| --- | --- |
 Reference:3 || Sentence | "Iron loading markedly reduced intracellular expression of ferroportin." |
| PMID | 14684575 |
| Year | 2003 |
| Species | Human |
|  | Rat |
| Journal | Gut |
| RefScore | 0 |
| Source | PArchNLP |
  ||  |  |


---

|  |  |
| --- | --- |
 Reference:4 || Sentence | "Circulating hepcidin then directly influences the expression of Ireg1 in the mature villus enterocytes of the duodenum, thereby regulating iron absorption in response to body iron requirements." |
| PMID | 12737947 |
| Year | 2003 |
| Species | Human |
| Journal | Blood Cells Mol Dis |
| RefScore | 1 |
| Source | PArchNLP |
  ||  |  |


---

|  |  |
| --- | --- |
 Reference:5 || Sentence | "The aim of this study was to investigate the effects of iron treatment on DMT1 and IREG1 mRNA expression in Caco-2 cells, a human intestinal cell line." |
| PMID | 11925462 |
| Year | 2002 |
| Species | Human |
| Journal | J Nutr |
| RefScore | 1 |
| Source | PArchNLP |
  ||  |  |


---

|  |  |
| --- | --- |
 Reference:6 || Sentence | "Interestingly, exposure to copper increased iron efflux from Caco-2 cells and up regulated IREG1 (iron-regulated mRNA) expression." |
| PMID | 12220667 |
| Year | 2002 |
| Species | Human |
| Journal | FEBS Lett |
| RefScore | 1 |
| Source | PArchNLP |
  ||  |  |


---

|  |  |
| --- | --- |
 Reference:7 || Sentence | "The iron chelator pyrrolidinedithiocarbamate (PDTC) stimulated hepatic hepcidin without suppressing intestinal FPT1 expression." |
| PMID | 14592944 |
| Year | 2003 |
| Species | Rat |
| Journal | Am J Physiol Gastrointest Liver Physiol |
| RefScore | 0 |
| Source | PArchNLP |
  ||  |  |


---

|  |  |
| --- | --- |
 Reference:8 || Sentence | "On day 10, no significant effect of iron supplementation on DMT1 and FPN1 gene expression or on iron absorption was observed." |
| PMID | 14668284 |
| Year | 2003 |
| Species | Human |
|  | Rat |
| Journal | Am J Clin Nutr |
| RefScore | 1 |
| Source | PArchNLP |
  ||  |  |


---

|  |  |
| --- | --- |
 Reference:9 || Sentence | "Iron deficiency induces MTP1 expression in the duodenum but down-regulates expression in the liver." |
| PMID | 10747949 |
| Year | 2000 |
| Species | Mouse |
|  | Human |
| Journal | J Biol Chem |
| RefScore | 0 |
| Source | PArchNLP |
  ||  |  |


---

|  |  |
| --- | --- |
 Reference:10 || Sentence | "Body iron status regulates ferroportin-1 (FPN1) expression such that intestinal mRNA levels are enhanced by anemia, whereas liver transcripts are increased by iron overload." |
| PMID | 14988440 |
| Year | 2004 |
| Species | Mouse |
| Journal | J Nutr |
| RefScore | 2 |
| Source | PArchNLP |
  |


---

|  |  |
| --- | --- |
